# Quaking-5 suppresses aggressiveness of lung cancer cells through inhibiting β-catenin signaling pathway

**DOI:** 10.18632/oncotarget.19066

**Published:** 2017-07-07

**Authors:** Xuexia Zhou, Xuebing Li, Cuiyun Sun, Cuijuan Shi, Dan Hua, Lin Yu, Yanjun Wen, Feng Hua, Qian Wang, Qinghua Zhou, Shizhu Yu

**Affiliations:** ^1^ Department of Neuropathology, Tianjin Key Laboratory of Injuries, Variations and Regeneration of The Nervous System, Key Laboratory of Post-Trauma Neuro-Repair and Regeneration in Central Nervous System of Education Ministry, Tianjin Neurological Institute, Tianjin Medical University General Hospital, Tianjin 300052, China; ^2^ Tianjin Key Laboratory of Lung Cancer Metastasis and Tumor Microenvironment, Tianjin Lung Cancer Institute, Tianjin Medical University General Hospital, Tianjin 300052, China; ^3^ Department of Biochemistry and Molecular Biology, School of Basic Medical Sciences of Tianjin Medical University, Tianjin 300070, China; ^4^ Department of Surgery, Shandong Cancer Hospital and Institute, Jinan 250117, China

**Keywords:** QKI-5, lung cancer, pro-metastasis processes, β-catenin, methylation

## Abstract

Quaking-5 (QKI-5) belongs to the STAR (signal transduction and activation of RNA) family of RNA binding proteins and functions as a tumor suppressor in several human malignancies. In this study, we attempt to elucidate the role of QKI-5 in the pro-metastasis processes of lung cancer (LC) cells and the underlying mechanisms. We confirmed that QKI-5 was decreased in human LC tissues and cell lines, especially in high-metastatic cells. Moreover, QKI expression was positively correlated with LC patients’ survival. Functional studies verified that QKI-5 suppressed migration, invasion and TGF-β1-induced epithelial-mesenchymal transition (EMT) of LC cells. Mechanistically, we affirmed that QKI-5 reduced β-catenin level in LC cells via suppressing its translation and promoting its degradation, whereas QKI-5 promoter hypermethylation suppressed QKI-5 expression. Our findings indicate that QKI-5 inhibits pro-metastasis processes of LC cells through interdicting β-catenin signaling pathway, and that QKI-5 promoter hypermethylation is a crucial epigenetic regulation reducing QKI-5 expression in LC cells, and reveal that QKI-5 is a potential prognostic biomarker for LC patients.

## INTRODUCTION

Lung cancer (LC) currently ranks as one of the most prevalent neoplasms and the leading cause of cancer-related deaths worldwide [1]. According to the histological characters, LC can be classified into small cell lung carcinoma (SCLC) and non-small cell lung carcinoma (NSCLC) which accounts for about 85% of all the LCs [2]. Confronting the facts of its increasing incidence, considerable mortality and poor prognosis, LC has become a great threat for human health [3]. Therefore, further efforts are urgently needed to comprehensively understand the internal biological mechanisms and to identify more candidate therapy targets for improving the clinical outcomes of LC patients.

As is known, metastasis occurs in about 40% of newly diagnosed LC patients [4]. For all types of LCs, treatment failure is mainly attributed to distant metastasis involving lymph node, brain and liver [5]. Epithelial-mesenchymal transition (EMT) is a biological process that enables epithelial cells to acquire mesenchymal phenotype [6]. It can be triggered by various ligand-receptor interactions, including TGF-β1, and involves extensive regulatory networks which are controlled by transcription factors and else [7]. Over the past decades, increasing evidences have demonstrated that EMT contributes to tumor progression, especially to tumor invasion and metastasis [6]. Wnt/β-catenin signaling pathway is crucial in human malignancies, including LC [8]. Notably, numerous studies have linked the metastasis and EMT induction with Wnt/β-catenin activation [9–11]. Hence, it is important to understand how the crosstalk between key factors and Wnt/β-catenin signaling pathway promotes the aggression of LC cells, which may provide new modalities for LC therapeutic intervention.

Quaking (QKI), a member of signal transduction and activation of RNA (STAR) protein family, is a pivotal post-transcriptional regulator [12]. The *QKI* gene encodes three major alternatively spliced mRNAs, i.e., *QKI-5*, *QKI-6*, and *QKI-7* with different C-terminals. QKI-5 is prominently expressed in early embryogenesis, while QKI-6 and -7 are mainly expressed in the central nervous system in late development stage [13]. Through recognition and binding to the QKI responsive element (QRE, ACUAAY_[N1–20]_UAAY) in the 3′-UTR of mRNA, QKI impacts diverse aspects of target mRNAs including the location, stability, and translational efficiency in multiple physiological and pathological processes [14]. In LC, it has been demonstrated that QKI-5 is the dominant isoform of QKIs [15], and its downexpression is an important cause leading to cell proliferation acceleration by influencing alternative mRNA splicing of at least two genes, *NUMB* and *MacroH2A1* [15, 16]. However, the effect of QKI-5 on LC metastasis and the underlying mechanism remain poorly understood.

In the present study, we confirm that QKI-5 is decreased in human LC tissues and cell lines, especially in high-metastatic cells, and identify that QKI-5 inhibits the migration, invasion and TGF-β1-induced EMT of LC cells by directly decreasing β-catenin. Moreover, we uncover that QKI-5 reduction in LC cells results from *QKI* promoter hypermethylation. Our data demonstrate for the first time that QKI-5 is a key inhibitor of LC cell aggressiveness and that methylation of *QKI* promoter contributes to QKI-5 downexpression in LC.

## RESULTS

### QKI-5 expression is decreased in high-metastatic LC tissues and cell lines

**Figure 1:**
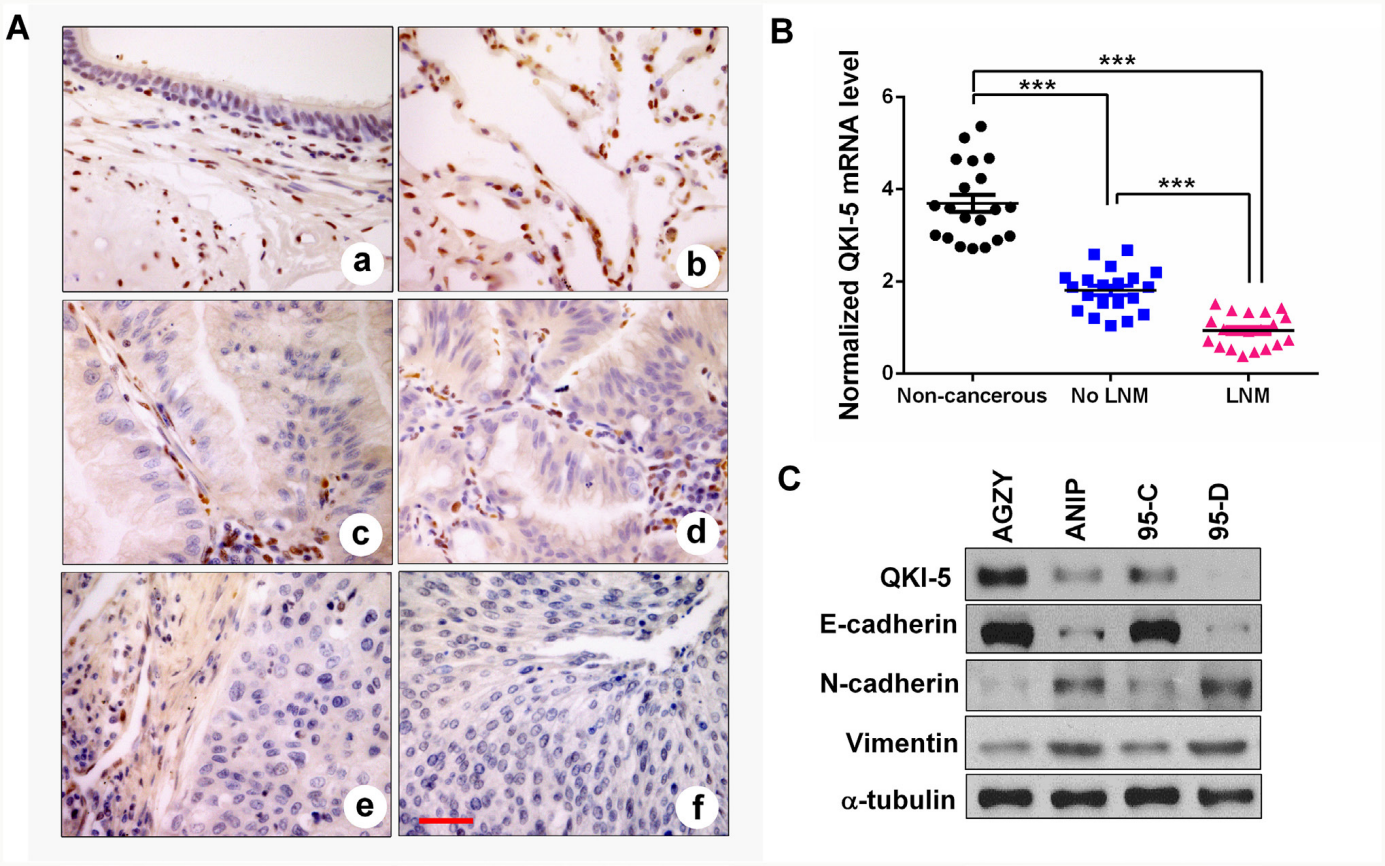
QKI-5 downexpression in human LC tissues and cell lines with metastasis is accompanied by EMT **(A)** IHC representative images of QKI-5 expression in bronchial epithelial **(a)**, alveolar epithelium **(b)**, lung adenocarcinoma **(c, d)** and lung squamous cancer **(e, f)**. Scale bar, 50 μm. **(B)** Comparisons among groups of *QKI-5* mRNA level in 20 non-cancerous lung tissues, 20 LC tissues without (No LNM) and 20 LC tissues with (LNM) lymph node metastasis. The *QKI-5* mRNA level was analyzed by qRT-PCR and normalized against *GAPDH*. The ratio of *QKI-5*/*GAPDH* in LNM group was arbitrarily set to 1.0. The data are presented as the mean ± SD. ***P<0.001. **(C)** Western blotting analyses of QKI-5 and EMT markers (E-cadherin, N-cadherin, Vimentin) in two pairs of differential metastatic LC cell lines (AGZY vs ANIP, 95C vs 95D). α-tubulin was used as the loading control.

To investigate the potential roles of QKI in LC progression, we firstly characterized the expressions of several QKI isoforms generated by alternative splicing, and found that QKI-5 was the dominant isoform expressed in LC cells (Supplementary Figure 1A-1C). By utilizing the specific antibody as determined in Supplementary Figure 1C, we analyzed QKI-5 expression in LC tissues by IHC. The results showed that QKI-5 was homogeneously presented in the nucleus of bronchial epithelium and pneumocytes in non-cancerous lung tissues, but was nearly absent in lung adenocarcinoma and squamous cell carcinoma (Figure 1A). Significantly, the survival analysis of 1926 LC patients based on Kaplan Meier plotter demonstrated that lower *QKI* mRNA expression predicted a shorter overall survival (*P*<0.001; Supplementary Figure 2). The result reveals that QKI-5 is a potential prognostic biomarker for LC patients. We next detected *QKI-5* mRNA expression in 60 tissue samples (20 non-cancerous lung tissues, 20 LC samples without (No LNM) and 20 LC samples with lymph node metastasis (LNM)). In comparison with non-cancerous lung tissues, *QKI-5* mRNA reduction was more obvious in metastatic tumors than those of non-metastatic tumors (*P*<0.001; Figure 1B). We then examined the expressions of QKI-5 mRNA and protein in two pairs of low and high metastatic LC cell lines (AGZY vs ANIP, 95C vs 95D). As shown in Supplementary Figure 3A, *QKI-5* mRNA was significantly reduced in high-metastatic ANIP and 95D cells (*P*<0.05∼0.01), which was accompanied by the decrease of QKI-5 protein and E-cadherin as an epithelial marker, and increase of N-cadherin and Vimentin as mesenchymal markers (Figure 1C). These data indicate that QKI-5 is an inhibitor of LC metastasis and that its downexpression is an important cause inducing EMT and metastasis of LC.

### QKI-5 inhibits the migration and invasion of LC cells

**Figure 2:**
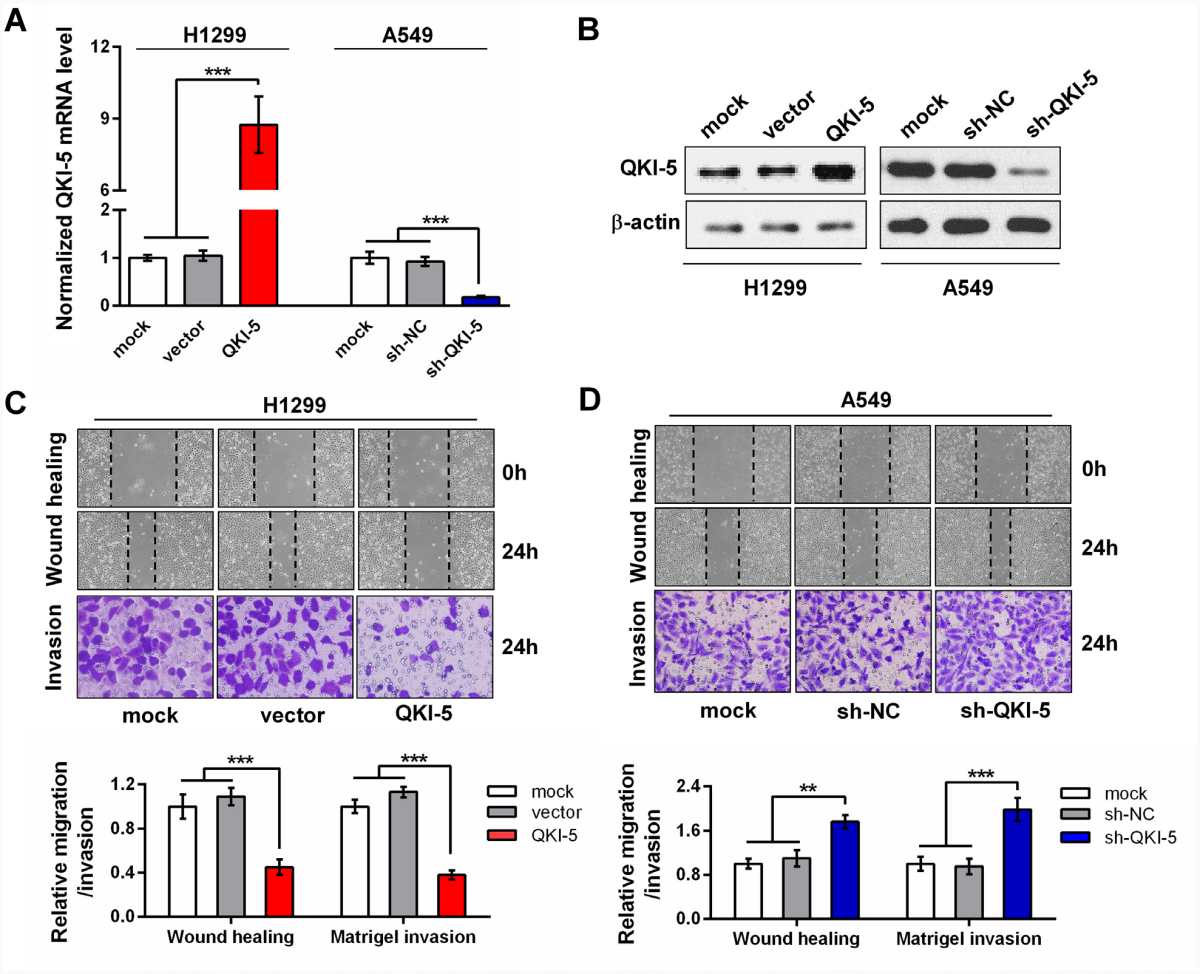
The inhibitory effects of QKI-5 on the migration and invasion of LC cells **(A** and **B)** Detections of mRNA **(A)** and protein **(B)** of QKI-5 overexpression and knockdown efficiencies in stable H1299 and A549 subcell lines constructed with indicated viruses or plasmids. The *QKI-5* mRNA level was analyzed by qRT-PCR and normalized against *GAPDH*. The ratios of *QKI-5*/*GAPDH* in the mock groups were arbitrarily set to 1.0. **(C** and **D)** Representative images (upper) and quantitative data (bottom) of migration and invasion of the indicated H1299 **(C)** and A549 **(D)** subcell lines assessed by wound-healing and transwell assays. All the experiments were performed at least in triplicate and the data in A, C and D are presented as the mean ± SD. **P<0.01, ***P<0.001.

To elucidate the possible roles of QKI-5 in the pro-metastasis processes of LC cells, we examined its expression level in multiple LC cell lines. The results showed that QKI-5 was decreased in these LC cell lines compared with a bronchial epithelial cell (Beas2B), accompanied by the increase of N-cadherin and Vimentin (Supplementary Figure 3B). As shown in Supplementary Figure 3B, QKI-5 level was the lowest in H1299 cells and modest in A549 cells. Therefore, gain-of-function studies were performed in H1299 cells, whereas loss-of-function studies in A549 cells. Stable QKI-5-overexpressing H1299 cells and QKI-5-silenced A549 cells were constructed by respective retroviruses or plasmids (Figure 2A and 2B). Wound healing and transwell invasion assays showed that QKI-5 overexpression significantly suppressed the migration and invasion of H1299 cells (*P*<0.001; Figure 2C). In contrast, QKI-5 knockdown facilitated the migration and invasion of A549 cells (*P*<0.01∼0.001; Figure 2D). Identical effects were also observed in the SPC-A1 cells (*P*<0.05∼0.001; Supplementary Figure 4A-4C). Since migration and invasion are the key procedures of LC metastasis, these findings further identify the inhibitory effect of QKI-5 on the pro-metastatic behaviors of LC cells.

### QKI-5 overexpression attenuates the TGF-β1-induced EMT of LC cells

**Figure 3:**
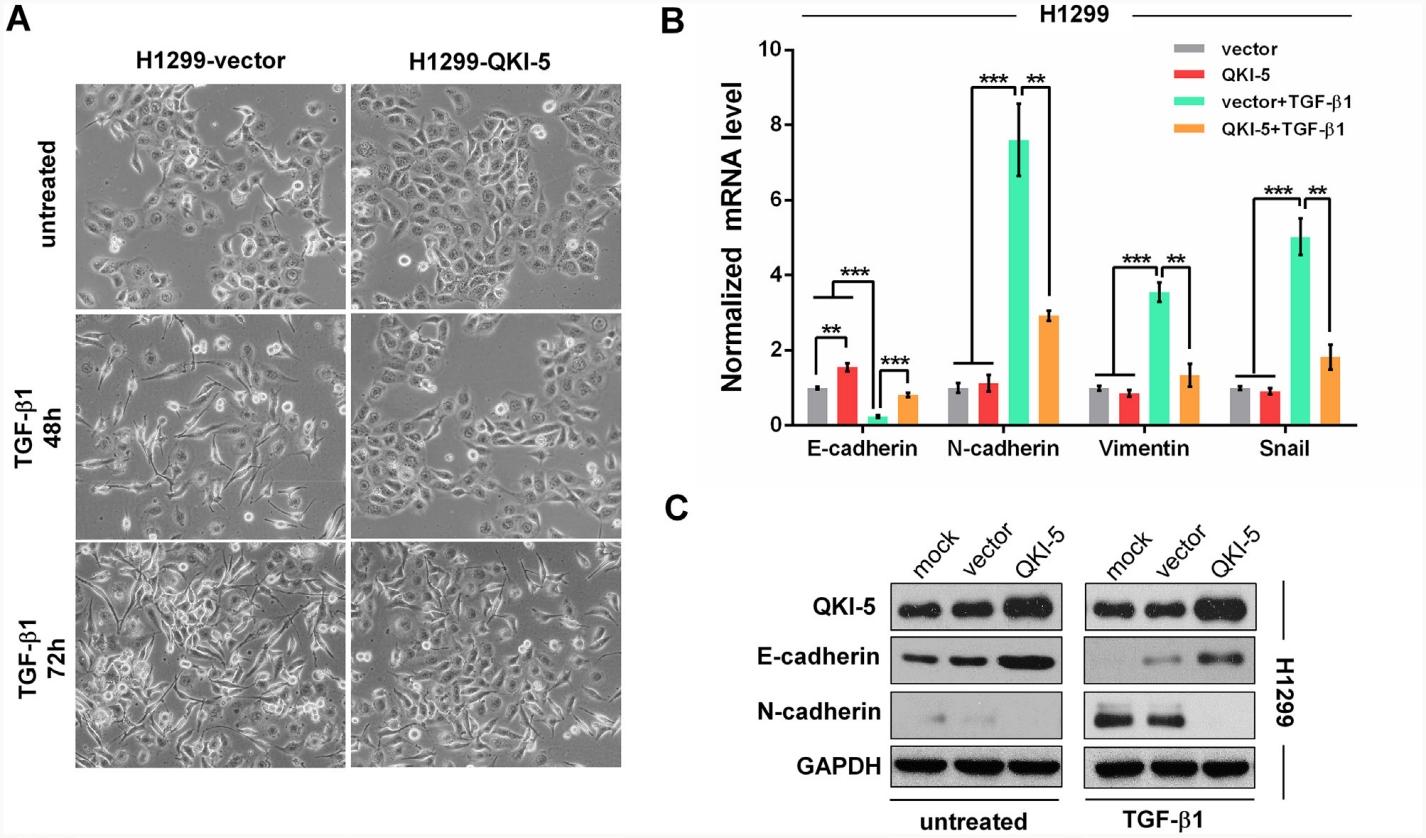
QKI-5 overexpression restrains the TGF-β1-induced EMT of LC cells **(A)** Representative images of H1299-vector (control) and H1299-QKI-5 (QKI-5-overexpressing) cells untreated or incubated with TGF-β1 (5 ng/mL) for 48 or 72 h (×200). **(B)** mRNA levels of *E-cadherin*, *N-cadherin*, *Vimentin* and *Snail* in the indicated cells incubated with TGF-β1 or untreated in DMEM for 72 h as in **A.** The mRNAs were detected by qRT-PCR and normalized against *GAPDH*. The ratios of the above mRNAs to *GAPDH* mRNA in control cells (vector) were arbitrarily set to 1.0. The data are presented as the mean ± SD. **P<0.01, ***P<0.001. **(C)** Protein levels of QKI-5, E-cadherin and N-cadherin measured by Western blotting in the indicated H1299 cells.

It has been previously reported that NSCLC H1299 cells are converted to fibroblastic phenotype in response to TGF-β1. To ascertain the role of QKI-5 in LC cell EMT, we conducted the experiment inducing EMT with TGF-β1 (5 ng/ml). At 48 h after induction, H1299-vector control cells showed spindle fibroblast-like morphology with reduced cell-cell contact and appeared more significant at 72 h, whereas QKI-5-overexpressing H1299-QKI-5 cells still remained epithelial shape at 48 h after induction and only exhibited minor morphological alteration at 72 h (Figure 3A). qRT-PCR and Western blotting analyses verified that TGF-β1-induced morphological transformation of H1299-vector cells was accompanied by significant decrease of E-cadherin as an epithelial marker and increase of N-cadherin, Vimentin, Snail as mesenchymal markers (*P*<0.001; Figure 3B and 3C). Moreover, QKI-5 overexpression in H1299-QKI-5 cells not only increased E-cadherin but also significantly reversed the suppressive or stimulative effect of TGF-β1 on the expression of E-cadherin, N-cadherin, Vimentin or Snail (*P*<0.01∼0.001; Figure 3B and 3C), which was consistent with their epithelial phenotype with enhanced cell-cell contact (Figure 3A). QKI-5 overexpression in A549 cells also induced similar resistance to TGF-β1-induced EMT (*P*<0.01∼0.001; Supplementary Figure 5A and 5B). These results verify that QKI-5 overexpression can indeed interdict TGF-β1-induced EMT of LC cells.

### QKI-5 inhibits LC cell invasion and EMT by blocking β-catenin signaling pathway

**Figure 4:**
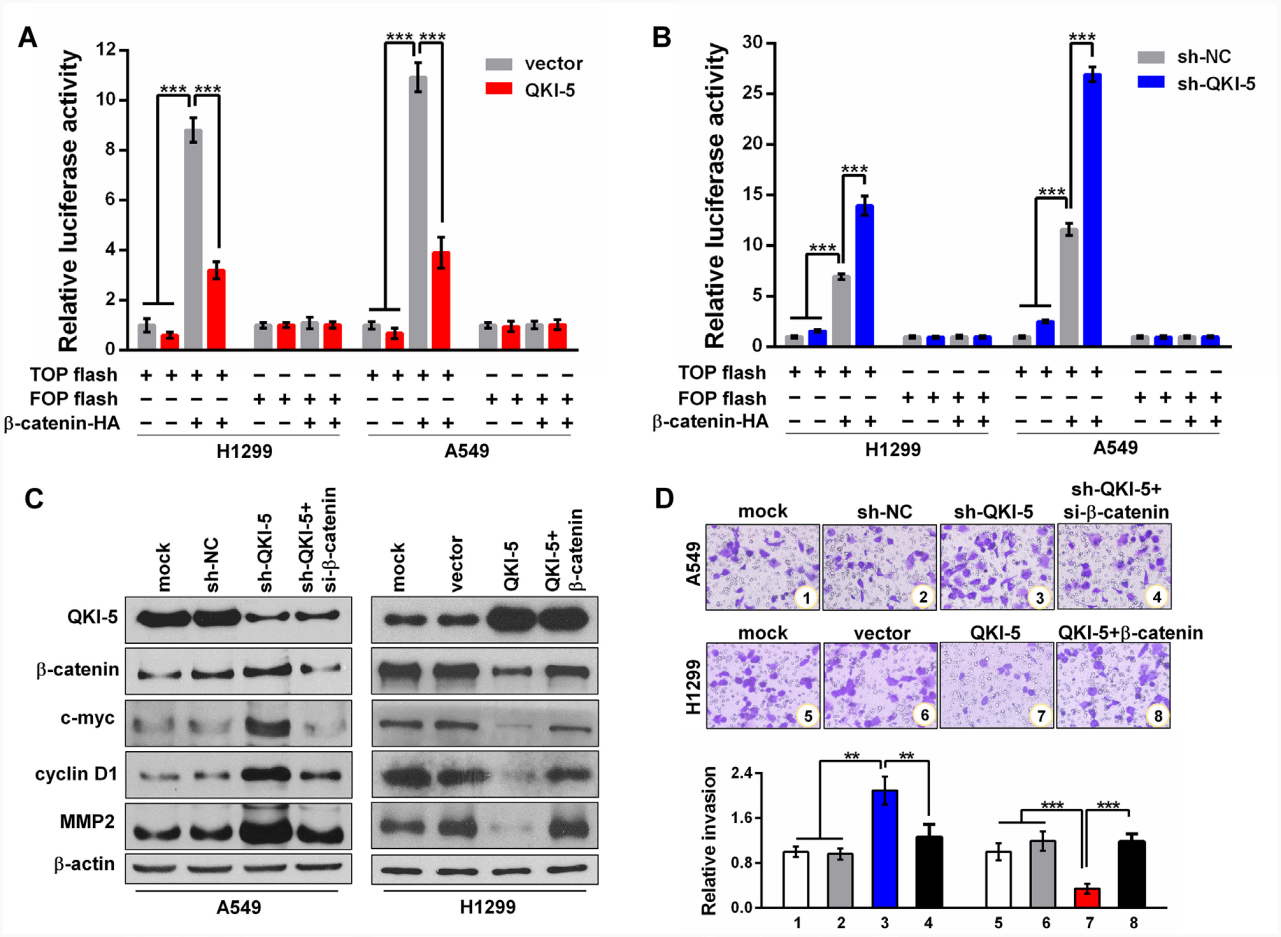
QKI-5 suppresses LC cell invasion by blocking β-catenin signaling pathway **(A** and **B)** Relative luciferase activities in subcell lines of control (H1299-vector, A549-vector) and QKI-5 expression (H1299-QKI-5, A549-QKI-5) **(A)**, or in subcell lines of control (H1299-sh-NC, A549-sh-NC) and QKI-5 knockdown (H1299-sh-QKI-5, A549-sh- QKI-5) **(B)**, co-transfected with TOP or FOP flash plus β-catenin-expressing plasmid (β-catenin-HA) as β-catenin/TCF/LEF pathway activator, and β-galactosidase-expressing plasmid as transfection efficiency control. TOP flash=the reporter plasmid containing three wild type binding sites of β-catenin-TCF/LEF complex in its *luciferase* promoter, FOP flash=the negative control plasmid containing the mutated binding sites of β-catenin-TCF/LEF complex. The data were normalized according to the ratio of firefly luciferase activity to the β-galactosidase value. **(C)** Protein levels of QKI-5, β-catenin, c-myc, cyclin D1 and MMP2 detected by Western blotting in the indicated cells. **(D)** Representative images (upper) and quantitative data (bottom) of invasion of the indicated A549 and H1299 cells assessed by transwell assay. All the experiments were performed at least in triplicate and the data in A, B and D are presented as the mean ± SD. **P<0.01, ***P<0.001.

β-catenin functions as a transcription factor by forming β-catenin-TCF/LEF complex and its mRNA is a target of QKI. To determine whether the anti-invasion effects of QKI-5 on LC cells involve the β-catenin-activated transcriptions of downstream genes, we performed TOP/FOP flash reporter assays and found that QKI-5-overexpression significantly reduced, whereas QKI-5-knockdown increased the β-catenin-activated luciferase activity of TOP flash in both H1299 and A549 cells (*P*<0.001; Figure 4A and 4B). It has been known that *c-myc*, *cyclin D1* and *MMP2* are the genes activated by β-catenin. Western blotting verified that β-catenin, c-myc, cyclin D1 and MMP2 were synchronously increased in QKI-5-silenced A549 cells, and decreased in QKI-5-overexpressing H1299 cells, while β-catenin knockdown or overexpression could effectively reverse the above effects of QKI-5 knockdown or overexpression on c-myc, cyclin D1 and MMP2 expressions (Figure 4C). Meanwhile, the enhanced A549 cell invasion by QKI-5 knockdown could be suppressed by β-catenin knockdown, and the inhibited H1299 cell invasion by QKI-5 overexpression could be regained by β-catenin overexpression (*P*<0.01∼0.001; Figure 4D). Furthermore, β-catenin overexpression rescued the TGF-β1-induced EMT of QKI-5-overexpressing H1299 cells, whereas β-catenin knockdown attenuated the TGF-β1-induced EMT of QKI-5-silenced A549 cells (Supplementary Figure 5C). These results identify that QKI-5 inhibits the invasion and TGF-β1-induced EMT of LC cells by blocking β-catenin signaling pathway.

### QKI-5 promotes β-catenin degradation and represses its translation in LC cells

**Figure 5:**
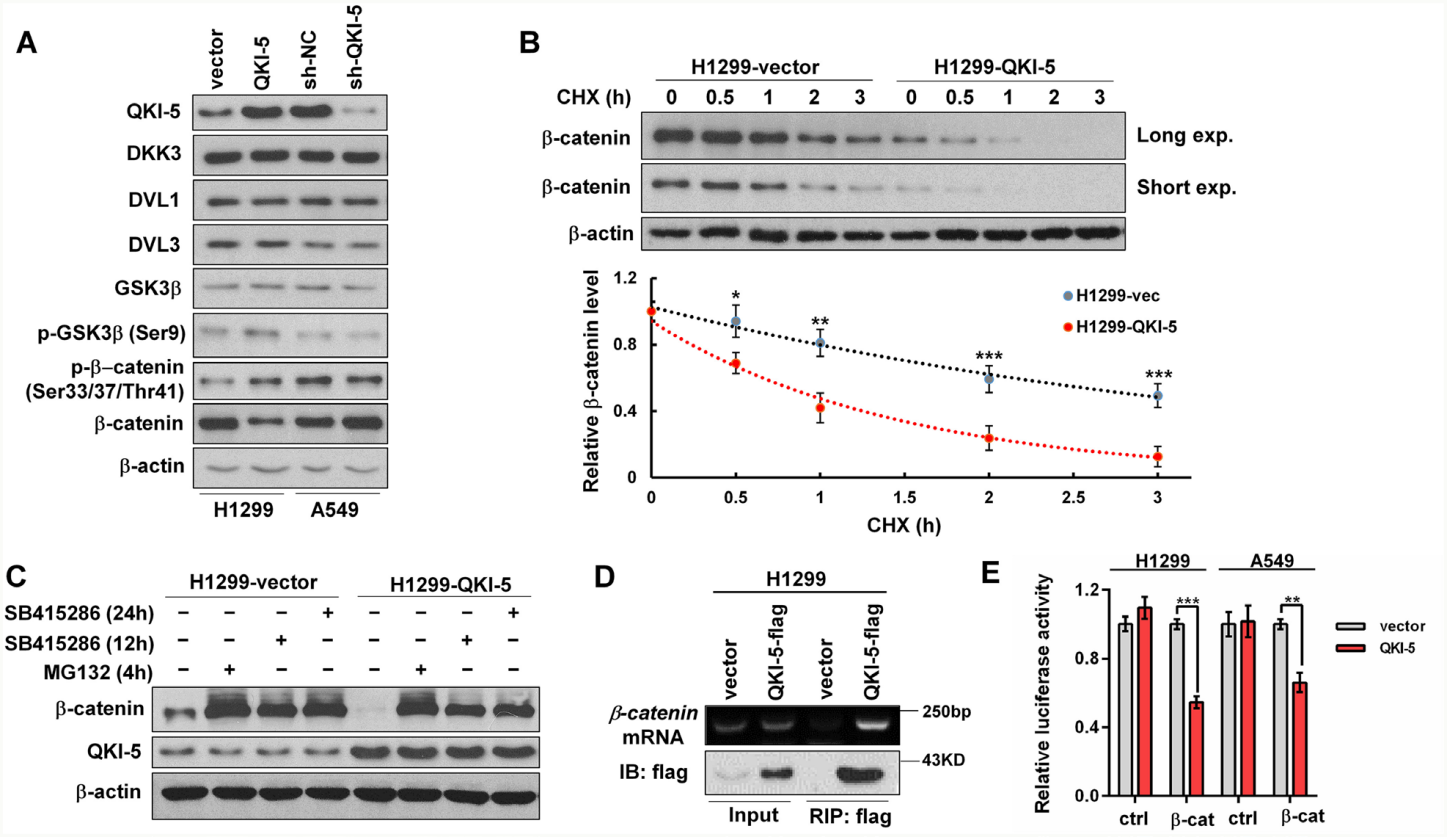
The mechanisms by which QKI-5 decreases β-catenin in LC cells **(A)** Western blotting analyses of QKI-5, DKK3, DVL1, DVL3, GSK3β, p-GSK3β (Ser9), p-β-catenin (Ser33/37/Thr41) and β-catenin in the indicated cells. **(B)** Comparison of β-catenin degradation between control (H1299-vector) and QKI-5-overexpressing (H1299-QKI-5) cells in which β-catenin synthesis was interdicted with CHX (100 μg/mL). The β-catenin levels at the indicated time points were detected by Western blotting (upper) and normalized against 0 h (bottom). **(C)** Comparisons of β-catenin and QKI-5 levels between H1299-vector and H1299-QKI-5 cells in which β-catenin degradation was blocked with proteasome inhibitor MG132 or GSK3β inhibitor SB415286 (25μM). **(D)** Directly binding between QKI-5-flag and endogenous *β-catenin* mRNA verified by RIP. H1299 cells were transfected as indicated. 36 h later, the cell lysates were prepared and QKI-5-flag was immunoprecipitated using anti-flag-beads. The *β-catenin* mRNA in immunoprecipitation was detected by RT-PCR. **(E)** Dual luciferase reporter assays in H1299 and A549 cells cotransfected with ctrl or β-cat reporter and vector or QKI-5 expressing plasmid; ctrl=the empty reporter plasmid, β-cat=the reporter plasmid of wild-type β-catenin 3′-UTR. The data were normalized according to the ratio of firefly luciferase activity to renilla luciferase activity. All experiments were performed at least in triplicate and the data in B and E are presented as the mean ± SD. *P<0.05, **P<0.01, ***P<0.001.

As is known, GSK3β phosphorylated at Ser9 (p-GSK3β) can catalyze β-catenin phosphorylation at Ser33/Ser37/Thr41 (p-β-catenin) and initiates p-β-catenin ubiquitin- dependent degradation. Western blotting analyses showed that β-catenin was reduced or increased, but the levels of DKK3, DVL1, DVL3 and GSK3β, the key regulators of Wnt/β-catenin signaling pathway, were constant in QKI-5-overexpressing or -silenced LC cells (Figure 5A). In contrast to β-catenin, p-GSK3β (Ser9) and p-β-catenin (Ser33/37/Thr41) were increased in QKI-5-overexpressing H1299 cells and decreased in QKI-5-silenced A549 cells (Figure 5A). To further verify these findings, H1299-vector cells (control) and QKI-5-overexpressing H1299-QKI-5 cells were treated using cycloheximide (CHX), a translation elongation inhibitor. After new β-catenin synthesis was interdicted by CHX, β-catenin reduction in H1299-QKI-5 cells was significantly faster than that in H1299-vector cells (*P*<0.05∼0.001; Figure 5B). These results indicate that QKI-5 can accelerate β-catenin degradation by inducing GSK3β phosphorylation in LC cells.

As shown in Figure 5C, β-catenin accumulation in H1299-QKI-5 cells was still lower than that in H1299-vector cells when its degradation was blocked by GSK3β inhibitor (SB415286) or proteasome inhibitor (MG132), but both the overexpression and knockdown of QKI-5 did not change *β-catenin* mRNA transcription in two LC cells (Supplementary Figure 5D), suggesting that QKI-5 might also suppress β-catenin expression through inhibiting its translation. It is known that the 3’-UTR of *β-catenin* mRNA contains two potential QREs. RNA immunoprecipitation (RIP) confirmed that flag-tagged QKI-5 was co-precipitated with *β-catenin* mRNA in H1299 cells (Figure 5D). We then constructed the luciferase reporter containing the two potential QREs of *β-catenin* 3’-UTR, and verified that QKI-5 overexpression obviously inhibited the activity of *β-catenin* 3’-UTR in H1299 and A549 cells (*P*<0.01∼0.001; Figure 5E). The above results reveal that QKI-5 not only expedites β-catenin degradation, but also suppresses β-catenin translation by binding with the QREs in its mRNA 3’-UTR, thereby decreasing β-catenin and directly blocking its signaling pathway in LC cells.

### *QKI* promoter hypermethylation inhibits its expression in LC cells

**Figure 6:**
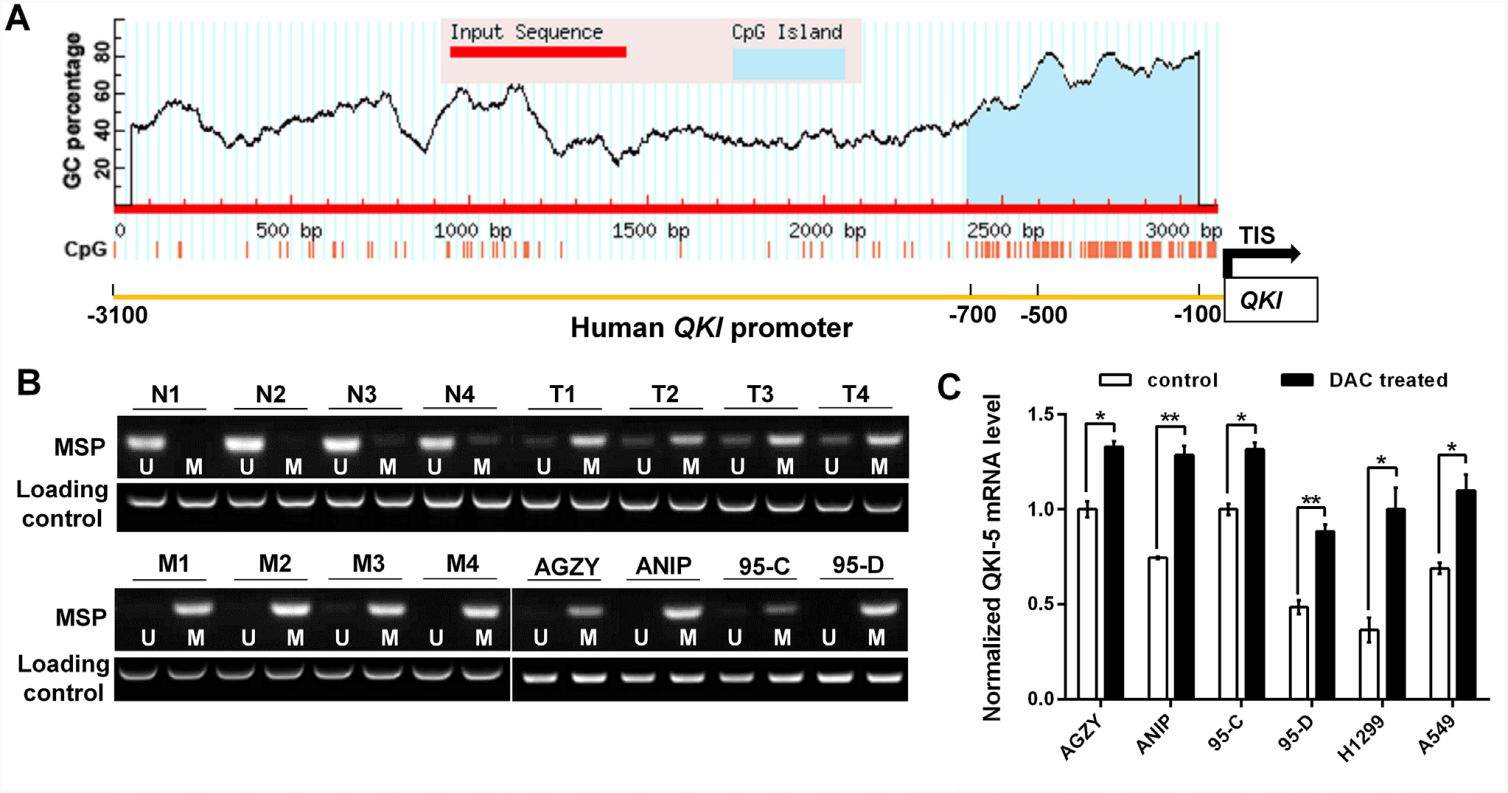
QKI promoter hypermethylation inhibits its expression in LC cells **(A)** CpG island predicted from -3100bp to transcription initiation site (TIS) in *QKI* promoter. **(B)** Methylation status of *QKI* promoter in non-cancerous lung tissues (N, n=4), LC tissues without LNM (T, n=4) and with LNM (M, n=4) from 12 individuals, and LC cell lines with lower (AGZY and 95C) or higher (ANIP and 95D) metastatic potency. The detection was executed by methylation specific PCR (MSP); U = unmethylated, M = methylated. **(C)** *QKI-5* mRNA level in the indicated cells analyzed by qRT-PCR and normalized against *GAPDH*. The ratio of *QKI-5*/*GAPDH* in control AGZY cells was arbitrarily set to 1.0. The data are presented as the mean ± SD. *P<0.05, **P<0.01.

To further clarify the underlying mechanism resulting in QKI-5 downexpression in LC, we focused on *QKI* promoter methylation. The online promoter analysis found that *QKI* promoter region, especially from -700bp to transcription initiation site (TIS), had abundant CpG islands (Figure 6A). Methylation specific PCR (MSP) verified that *QKI* promoter methylation was very frequent in LC and LC cell lines, and more obvious in LC with LNM and high-metastatic ANIP and 95-D cells (Figure 6B), which was in accord with the QKI-5 expression level in Figure 1B and 1C. Furthermore, the treatment of 5-aza-2′-deoxycytidine (DAC), a demethylation agent, significantly increased *QKI-5* mRNA level in LC cells (Figure 6C). The results indicate that *QKI-5* promoter hypermethylation is an important mechanism leading to QKI-5 downexpression in LC cells.

## DISCUSSION

QKI-5 is a vital regulator of RNA processing and cell signal transduction, and the change of alternative mRNA splicing induced by its downexpression is an important cause accelerating cell proliferation in multiple cancers including LC [15–17]. However, whether QKI-5 suppresses LC metastasis and the underlying mechanism remain unknown. In the present study, we identified that QKI-5 expression was decreased because of its promoter hypermethylation in LC, particularly in metastatic LC. Moreover, we uncovered for the first time its new functions suppressing migration, invasion and TGF-β1-induced EMT in LC through a novel mechanism decreasing β-catenin level in LC cells. These findings facilitated our understanding of LC metastatic mechanisms.

The relations between RNA-binding proteins including QKIs and cancers have attracted much attention [16, 18, 19]. Our results demonstrated that QKI-5 was the dominant isoform of QKIs in LC cells and that its expression in metastatic LC tissues and high-metastatic LC cell lines was lower than that in non-metastatic LC tissues and low-metastatic LC cell lines. Moreover, patients’ overall survival was positively correlated with *QKI* level. Furthermore, QKI-5 downexpression was accompanied the reduction of E-cadherin as an epithelial marker and the increase of N-cadherin and Vimentin as mesenchymal markers in high-metastatic LC cell lines. The functional assays confirmed that QKI-5 overexpression or knockdown could significantly suppress or facilitate the migration and invasion of LC cells, and that QKI-5 overexpression effectively inhibited the TGF-β1-induced EMT of LC cells. These facts indicated that QKI-5 was an important inhibitor of migration, invasion and EMT of LC cells, highlighting the accelerative effects of QKI-5 downexpression on LC pro-metastasis processes and revealing QKI-5 is a potential prognostic biomarker for LC patients.

It has been established that β-catenin, a key relay in Wnt signaling pathway, promotes the genesis and progression of LC via activating downstream gene transcription [20, 21]. Our results showed that QKI-5 overexpression or knockdown not only decreased or increased the luciferase activity of TOP flash receptor, and β-catenin accumulation and its downstream gene expressions (c-myc, cyclin D1, MMP2) in LC cells, but also suppressed or facilitated the invasion and TGF-β1-induced EMT of LC cells. Moreover, β-catenin overexpression or knockdown could effectively reverse the above effects of QKI-5 overexpression or knockdown. These data indicated that QKI-5 inhibited the migration, invasion and EMT of LC cells through blocking β-catenin signaling pathway, highlighting the potential values of QKI-5 and β-catenin in LC therapy.

Phosphorylation-activated p-GSK3β initiates the ubiquitin-dependent degradation of p-β-catenin by catalyzing β-catenin phosphorylation [22]. Our studies of QKI-5-overexpression, QKI-5-knockdown and β-catenin synthesis inhibition revealed that QKI-5 did not change the levels of DKK3, DVL1, DVL3 and GSK3β as Wnt/β-catenin signaling regulators in LC cells, but could accelerate the phosphorylation and ubiquitin-dependent degradation of β-catenin through increasing p-GSK3β, therefore decreasing β-catenin in LC cells. However, we found that the β-catenin accumulation in QKI-5-overexpressing H1299-QKI-5 cells was still lower than that in control H1299-vector cells after the above β-catenin degradation pathway was interdicted with GSK3β inhibitor (SB415286) or proteasome inhibitor (MG132), indicating that QKI-5 also decreased β-catenin by other mechanism in LC cells.

Previous studies have demonstrated that QKI-5 may suppress β-catenin translation by directly binding with QREs in *β-catenin* mRNA 3’-UTR [14, 23], and inhibits the proliferation of colon cancer cells by decreasing β-catenin [23]. In LC cells, we verified that QKI-5 could directly bind to QREs in *β-catenin* mRNA 3’-UTR and attenuate its translation efficiency by RNA immunoprecipitation and the 3’-UTR luciferase reporter, but did not reduce *β-catenin* mRNA. Our findings revealed that QKI-5 decreased β-catenin in LC cells by not only accelerating degradation but also repressing translation, thus blocking β-catenin signaling pathway in LC cells.

*QKI-5* promoter hypermethylation results in QKI-5 downexpression in colon cancer cells [23]. In the current study, we identified that *QKI* promoter region was rich in CpG islands, especially from -700bp to transcription initiation site. Moreover, *QKI* promoter was methylated in LC tissues and cell lines, and the methylation level was higher in metastatic LC tissues and high-metastatic LC cell lines. Furthermore, demethylation agent DAC significantly increased *QKI-5* expression in LC cells. These results indicate that *QKI-5* promoter hypermethylation is an important mechanism leading to QKI-5 downexpression in LC cells.

In summary, our study demonstrated that QKI-5 repressed the migration, invasion and TGF-β1-induced EMT of LC cells by directly decreasing β-catenin, and predicted better prognosis in LC patients. More importantly, we revealed a novel mechanism accelerating LC pro-metastatic processes, and QKI-5 and β-catenin might be the therapeutic candidates for this lethal disease.

## MATERIALS AND METHODS

### Tissue samples and clinical data

The surgical specimens of 40 LCs and 20 non-cancerous lung tissues (control) were collected from Tianjin Medical University General Hospital (TMUGH) with written consent. The metastatic status of patients was recorded on the basis of pathological and clinical examinations at the time of resection. The 40 LC samples were divided into two groups without or with LNM. The patients’ clinical features were summarized in Supplementary Table 1. For histological analysis, specimens were immediately fixed in 3.7% buffered formaldehyde solution after surgical excision and embedded in paraffin afterwards. Another repeats of tissues were stored in liquid nitrogen within 30 min after resection for detecting the mRNA and promoter methylation of *QKI-5*. This study was carried out in accordance with the principles of the Helsinki Declaration and approved by the Ethics Committee of TMUGH.

An independent cohort of 1926 LC patients from the Kaplan Meier plotter (http://www.kmplot.com) was used to verify the relevance of *QKI* mRNA level to overall survival of LC patients.

### Immunohistochemistry (IHC)

IHC staining was performed with rabbit anti-human QKI-5 primary antibody (Bethyl Laboratories, USA) as previouslydescribed [24]. IHC images were acquired under a DM6000B microscope (Leica, Germany).

### RNA isolation, RT-PCR and qRT-PCR

Total RNA was isolated using the TRIzol reagent (Invitrogen, USA) as the standard protocol. To determine the mRNA expressions of QKI transcripts, RT-PCR was performed as previously described [18], with M-MLV reverse transcriptase (Promega, USA). The specific primers were synthesized by BGI (Beijing Genomics Institute, China) and listed in Supplementary Table 2. qRT-PCR with the SYBR Green PCR kit (Takara Bio, China) was used to quantify the mRNAs involved in this study and *GAPDH* mRNA was used as the internal control. The specific primers were synthesized by BGI (China) and listed in Supplementary Table 3. The fold changes of mRNA levels were calculated by the 2^-ΔΔCt^ method.

### Western blotting

Western blotting was carried out as previously described [24]. The primary antibodies were purchased from Bethyl Laboratories (QKI), Cell Signaling Technology (E-cadherin, N-cadherin, Vimentin, Snail, Slug, p-β-catenin (Ser33/37/Thr41) and p-GSK3β (Ser9)), Santa Cruz Biotechnology (β-catenin, α-tubulin, β-actin, c-myc, cyclin D1, MMP2, DKK3, DVL1, DVL3, GSK3β), Boster (GAPDH) and Sigma (flag).

### Plasmid construction, virus package and stable subcell line screen

Human *QKI-5* cDNA sequence amplified by RT-PCR from the cDNA of H1299 cell line was cloned into pcDNA3.1 or pCMV-tag2b plasmid. The primers were listed in Supplementary Table 4. Then, *QKI-5* was subcloned into pMSCV-puro plasmid. The control (empty vector) or QKI-5-expressing retrovirus was constructed as previously described [25]. The infection of retroviral particles was performed as previously described [18]. Then, the stable subcell lines (H1299-vector, H1299-QKI-5, A549-vector, A549-QKI-5) of H1299 and A549 cells were established by selecting with puromycin (1 μg/mL; Sigma, USA). The plasmids expressing scramble sequences (sh-NC) and QKI-5 shRNA (sh-QKI-5) were constructed through inserting DNA fragments coding them into pENTR/H1/TO plasmid (Invitrogen, USA). The cells transfected with sh-NC or sh-QKI-5 were selected with zeocin (150 μg/mL; Sigma, USA). The sequences of shRNAs were listed in Supplementary Table 5.

### Cell culture and treatment

Human non-transformed bronchial epithelial cell line Beas2B, and LC cell lines H661, SPC-A1, SK-MES-1, H1299, H1373, H292 and A549 were all from the Chinese Infrastructure of Cell Line Resources. A549, H661, SPC-A1 and H292 cells were cultured in RPMI 1640 medium containing 10% FBS, and Beas2B, H1299, H1373 and SK-MES-1 cells were cultured in DMEM medium containing 10% FBS. All cell lines were maintained in an incubator containing 5% CO_2_ at 37°C. To induce EMT, H1299 and A549 cells were starved in medium containing 0.1% FBS for 16 h before treatment with 5 ng/mL TGF-β1 (ProSpec, Israel) for indicated hours. CHX (100 μg/mL; Sigma, USA) was used to treat H1299 cells for 0, 0.5, 1, 2, 3 h to interdict new β-catenin synthesis. To inhibit β-catenin degradation, H1299 cells were treated with proteasome inhibitor MG132 or GSK3β inhibitor SB415286 (25μM; MedChem Express, China) and harvested at 4 h for MG132 and 12 h or 24 h for SB415286.

### RNAi-mediated gene silencing

The small interfering RNA (siRNA) targeting β-catenin was synthesized by Gene Pharma (China), and the sequence was listed in Supplementary Table 6. The cells were transfected with 50 nM siRNA dissolved in DEPC water using Lipofectamine RNAiMAX (Invitrogen, USA) according to the manufacturer’s protocol.

### Wound healing and transwell invasion assays

Uniform artificial wounds were made at 2 d after transfection and the cells were cultured for another 24 h. Cell migration ability was represented by the wound gap distance in microscopic field (×40) at the time points of 0 and 24 h. For transwell invasion assay, at 24 h after transfection, the cells (3×10^4^/well) suspended in medium containing 1% FBS were seeded into the upper well of the transwell chamber pre-coated with 50 μl 1:4 diluted Matrigel (BD Bioscience, USA) and allowed to invade towards the medium containing 10% FBS for 24 h. The cells that reached the lower surface were fixed with methanol and stained with 0.1% crystal violet. The cells were counted in 5 randomly selected microscopic fields (×400) from each chamber.

### Luciferase reporter assays

The luciferase activity of TOP flash reporter carrying three optimal sites binding with β-catenin/TCF/LEF complex reflected the β-catenin-activated transcription activity of downstream gene. FOP flash reporter carrying the mutated binding sites was used as a negative control. The TOP/FOP flash assays were performed as previously described [26]. The subcell lines of control (H1299-vector, A549-vector, H1299-sh-NC, A549-sh-NC), QKI-5 expression (H1299-QKI-5, A549-QKI-5) and QKI-5 knockdown (H1299-sh-QKI-5, A549-sh- QKI-5) were co-transfected with TOP or FOP flash plus β-catenin-expressing plasmid (β-catenin-HA) as β-catenin/TCF/LEF pathway activator and β-galactosidase-expressing plasmid as transfection efficiency control using Lipofectamine 3000 (Invitrogen, USA). At 48 h after transfection, the luciferase and β-galactosidase activities in the lysates of these transfected cells were measured with the Luciferase Reporter Assay Kit (Promega, USA) and β-Gal Staining Kit (Invitrogen, USA). The results were presented as the luciferase activities normalized against those of β-galactosidase.

The luciferase reporter plasmid without (ctrl) or with wild type β-catenin 3’-UTR (β-cat) was structured as previously described [27]. H1299-vector, A549-vector, H1299-QKI-5 and A549-QKI-5 cells were transfected with ctrl or β-cat using Lipofectamine 3000 (Invitrogen, USA). At 24 h after transfection, the activities of firefly and renilla luciferases were measured as previously described [24, 27]. The results were presented as the firefly luciferase activities normalized against those of renilla.

### RNA immunoprecipitation (RIP)

RIP assay was performed using Magna RIP RNA-Binding Protein Immunoprecipitation Kit (Millipore, USA) according to manufacturer’s instruction. Flag antibody was used in RIP assays. The RNAs in immunoprecipitated complex and in the previously saved input fraction were extracted for further RT-PCR. Specific primers were applied for detecting the target mRNA (Supplementary Table 2).

### CpG island prediction

CpG island was predicted by the online Meth Primer tool (http://www.urogene.org/cgi-bin/methprimer/methprimer.cgi). The result was given at the default criteria (C+Gs/total bases >50%, CpG observed/expected >0.6).

### Methylation specific PCR (MSP)

Genomic DNA was extracted from LC tissues and cell lines using a DNA extraction kit (Promega, USA). After quantification, 2 μg of DNA was treated with sodium bisulfate as previously reported [28], and used as the template DNA for PCR. The MSP primers were designed with the MethPrimer (http://www.urogene.org/cgi-bin/methprimer/methprimer.cgi) [29] and sequences were listed in Supplementary Table 2. Then, the PCR products were electrophoresed on a 1.2% agarose gel.

### Statistical analyses

Statistical analyses were performed using SPSS 21.0 software (IBM, USA). One-way ANOVA test, Student *t* test, Pearson correlation analysis, Kaplan-Meier analysis and log-rank test were used to analyze corresponding data in this study. Results were presented as the mean ± standard deviation (SD). Statistical significance was assigned at *P*<0.05 (*), *P*<0.01 (**) or *P*<0.001 (***). All the experiments of cell lines were performed at least three times with triplicate samples.

## SUPPLEMENTARY MATERIALS FIGURES AND TABLES





## References

[1] Torre LA, Bray F, Siegel RL, Ferlay J, Lortet-Tieulent J, Jemal A (2015). Global cancer statistics, 2012. CA Cancer J Clin.

[2] Padda SK, Burt BM, Trakul N, Wakelee HA (2014). Early-stage non-small cell lung cancer: surgery, stereotactic radiosurgery, and individualized adjuvant therapy. Semin Oncol.

[3] Jemal A, Bray F, Center MM, Ferlay J, Ward E, Forman D (2011). Global cancer statistics. CA Cancer J Clin.

[4] Morgensztern D, Ng SH, Gao F, Govindan R (2010). Trends in stage distribution for patients with non-small cell lung cancer: a National Cancer Database survey. J Thorac Oncol.

[5] D'Antonio C, Passaro A, Gori B, Del Signore E, Migliorino MR, Ricciardi S, Fulvi A, de Marinis F (2014). Bone and brain metastasis in lung cancer: recent advances in therapeutic strategies. Ther Adv Med Oncol.

[6] Kalluri R, Weinberg RA (2009). The basics of epithelial-mesenchymal transition. J Clin Invest.

[7] Lamouille S, Xu J, Derynck R (2014). Molecular mechanisms of epithelial-mesenchymal transition. Nat Rev Mol Cell Biol.

[8] Stewart DJ (2014). Wnt signaling pathway in non-small cell lung cancer. J Natl Cancer Inst.

[9] Bernaudo S, Salem M, Qi X, Zhou W, Zhang C, Yang W, Rosman D, Deng Z, Ye G, Yang B, Vanderhyden B, Wu Z, Peng C (2016). Cyclin G2 inhibits epithelial-to-mesenchymal transition by disrupting Wnt/beta-catenin signaling. Oncogene.

[10] Douchi D, Ohtsuka H, Ariake K, Masuda K, Kawasaki S, Kawaguchi K, Fukase K, Oikawa M, Motoi F, Naitoh T, Katayose Y, Egawa S, Unno M (2015). Silencing of LRRFIP1 reverses the epithelial-mesenchymal transition via inhibition of the Wnt/β-catenin signaling pathway. Cancer Lett.

[11] Tong X, Li L, Li X, Heng L, Zhong L, Su X, Rong R, Hu S, Liu W, Jia B, Liu X, Kou G, Han J (2014). SOX10, a novel HMG-box-containing tumor suppressor, inhibits growth and metastasis of digestive cancers by suppressing the Wnt/beta-catenin pathway. Oncotarget.

[12] Kondo T, Furuta T, Mitsunaga K, Ebersole TA, Shichiri M, Wu J, Artzt K, Yamamura K, Abe K (1999). Genomic organization and expression analysis of the mouse qkI locus. Mamm Genome.

[13] Ebersole TA, Chen Q, Justice MJ, Artzt K (1996). The quaking gene product necessary in embryogenesis and myelination combines features of RNA binding and signal transduction proteins. Nat Genet.

[14] Galarneau A, Richard S (2005). Target RNA motif and target mRNAs of the Quaking STAR protein. Nat Struct Mol Biol.

[15] Zong FY, Fu X, Wei WJ, Luo YG, Heiner M, Cao LJ, Fang Z, Fang R, Lu D, Ji H, Hui J (2014). The RNA-binding protein QKI suppresses cancer-associated aberrant splicing. PLoS Genet.

[16] Novikov L, Park JW, Chen H, Klerman H, Jalloh AS, Gamble MJ (2011). QKI-mediated alternative splicing of the histone variant MacroH2A1 regulates cancer cell proliferation. Mol Cell Biol.

[17] Zhao Y, Zhang G, Wei M, Lu X, Fu H, Feng F, Wang S, Lu W, Wu N, Lu Z, Yuan J (2014). The tumor suppressing effects of QKI-5 in prostate cancer: a novel diagnostic and prognostic protein. Cancer Biol Ther.

[18] Zhou X, Li X, Cheng Y, Wu W, Xie Z, Xi Q, Han J, Wu G, Fang J, Feng Y (2014). BCLAF1 and its splicing regulator SRSF10 regulate the tumorigenic potential of colon cancer cells. Nat Commun.

[19] Busa R, Paronetto MP, Farini D, Pierantozzi E, Botti F, Angelini DF, Attisani F, Vespasiani G, Sette C (2007). The RNA-binding protein Sam68 contributes to proliferation and survival of human prostate cancer cells. Oncogene.

[20] Kim M, Suh YA, Oh JH, Lee BR, Kim J, Jang SJ (2016). KIF3A binds to beta-arrestin for suppressing Wnt/beta-catenin signalling independently of primary cilia in lung cancer. Sci Rep.

[21] Gong L, Song J, Lin X, Wei F, Zhang C, Wang Z, Zhu J, Wu S, Chen Y, Liang J, Fu X, Lu J, Zhou C (2016). Serine-arginine protein kinase 1 promotes a cancer stem cell-like phenotype through activation of Wnt/beta-catenin signalling in NSCLC. J Pathol.

[22] Rayasam GV, Tulasi VK, Sodhi R, Davis JA, Ray A (2009). Glycogen synthase kinase 3: more than a namesake. Br J Pharmacol.

[23] Yang G, Fu H, Zhang J, Lu X, Yu F, Jin L, Bai L, Huang B, Shen L, Feng Y, Yao L, Lu Z (2010). RNA-binding protein quaking, a critical regulator of colon epithelial differentiation and a suppressor of colon cancer. Gastroenterology.

[24] Liu J, Xu J, Li H, Sun C, Yu L, Li Y, Shi C, Zhou X, Bian X, Ping Y, Wen Y, Zhao S, Xu H (2015). miR-146b-5p functions as a tumor suppressor by targeting TRAF6 and predicts the prognosis of human gliomas. Oncotarget.

[25] Jang J, Yoon K, Hwang DW, Lee DS, Kim S (2012). A retroviral vector suitable for ultrasound image-guided gene delivery to mouse brain. Gene Ther.

[26] Deng YZ, Yao F, Li JJ, Mao ZF, Hu PT, Long LY, Li G, Ji XD, Shi S, Guan DX, Feng YY, Cui L, Li DS (2012). RACK1 suppresses gastric tumorigenesis by stabilizing the beta-catenin destruction complex. Gastroenterology.

[27] Li H, Yu L, Liu J, Bian X, Shi C, Sun C, Zhou X, Wen Y, Hua D, Zhao S, Ren L, An T, Luo W (2017). miR-320a functions as a suppressor for gliomas by targeting SND1 and beta-catenin, and predicts the prognosis of patients. Oncotarget.

[28] Ward AK, Mellor P, Smith SE, Kendall S, Just NA, Vizeacoumar FS, Sarker S, Phillips Z, Alvi R, Saxena A, Vizeacoumar FJ, Carlsen SA, Anderson DH (2016). Epigenetic silencing of CREB3L1 by DNA methylation is associated with high-grade metastatic breast cancers with poor prognosis and is prevalent in triple negative breast cancers. Breast Cancer Res.

[29] Li LC, Dahiya R (2002). MethPrimer: designing primers for methylation PCRs. Bioinformatics.

