# Supplementary material for: Quaking-5 suppresses aggressiveness of lung cancer cells through inhibiting β-catenin signaling pathway
Source: Oncotarget. 2017 Jul 7;8(47):82174–84. doi: 10.18632/oncotarget.19066 (PMC5669880; doi:10.18632/oncotarget.19066)
Supplement: Supplementary file 1 [file oncotarget-08-82174-s001.pdf]

Quaking-5 suppresses aggressiveness of lung cancer cells through inhibiting β-catenin signaling pathway

SUPPLEMENTARY MATERIALS

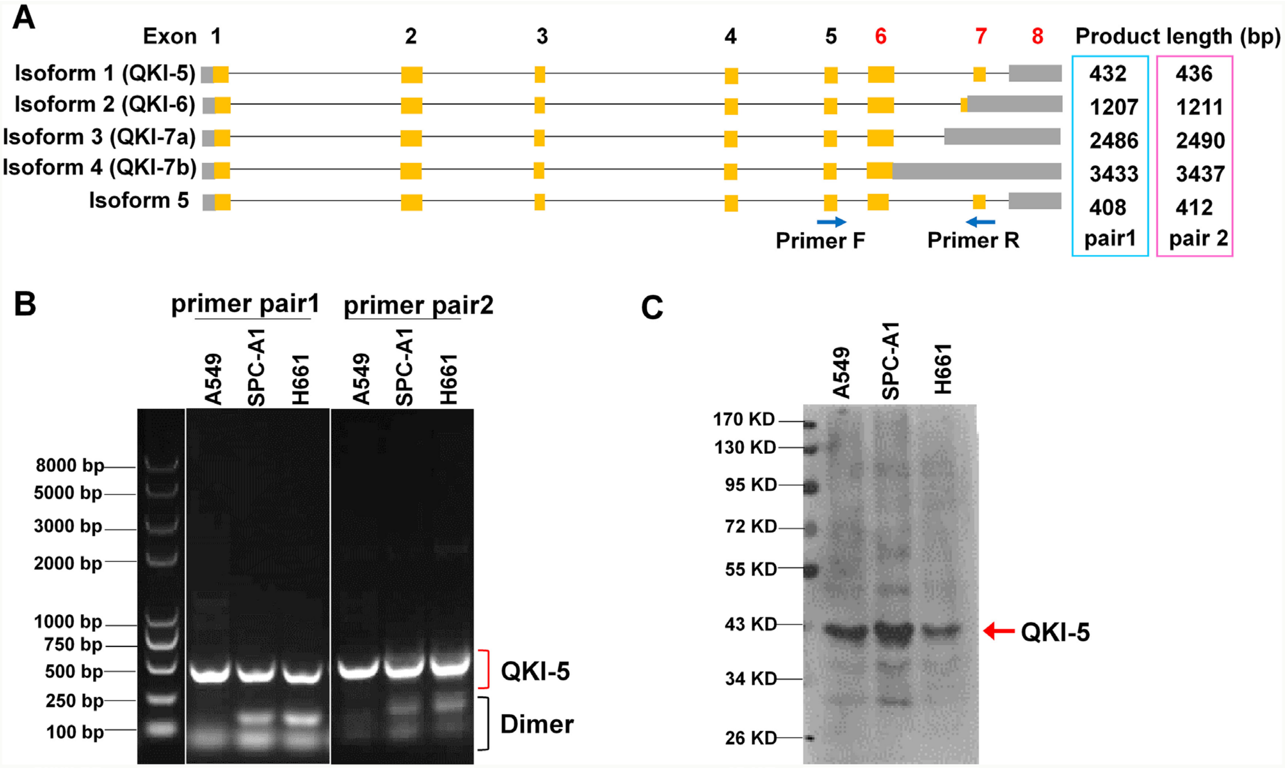

Supplementary Figure 1: QKI-5 is the dominant isoform of QKIs expressed in LC cells. **(A)** Schematic representation of the exon organization of five *QKI* isoforms generated by alternative splicing of exon6-exon8 annotated by NCBI. Gray boxes indicate 5' or 3' untranslated regions. Two pairs of RT–PCR primers flanking exons 6 and 7 (blue arrows) theoretically produce five splicing isoforms with respective length. **(B)** *QKI* mRNA splicing pattern in LC cell lines (A549, SPC-A1, H661) analyzed by RT–PCR using two primer pairs (blue arrows in **A**). Ful-gels showed that *QKI-5* was the dominant isoform. **(C)** Western blotting detection of QKI protein expression in the indicated LC cells. The results showed that QKI-5 was the dominant isoform.

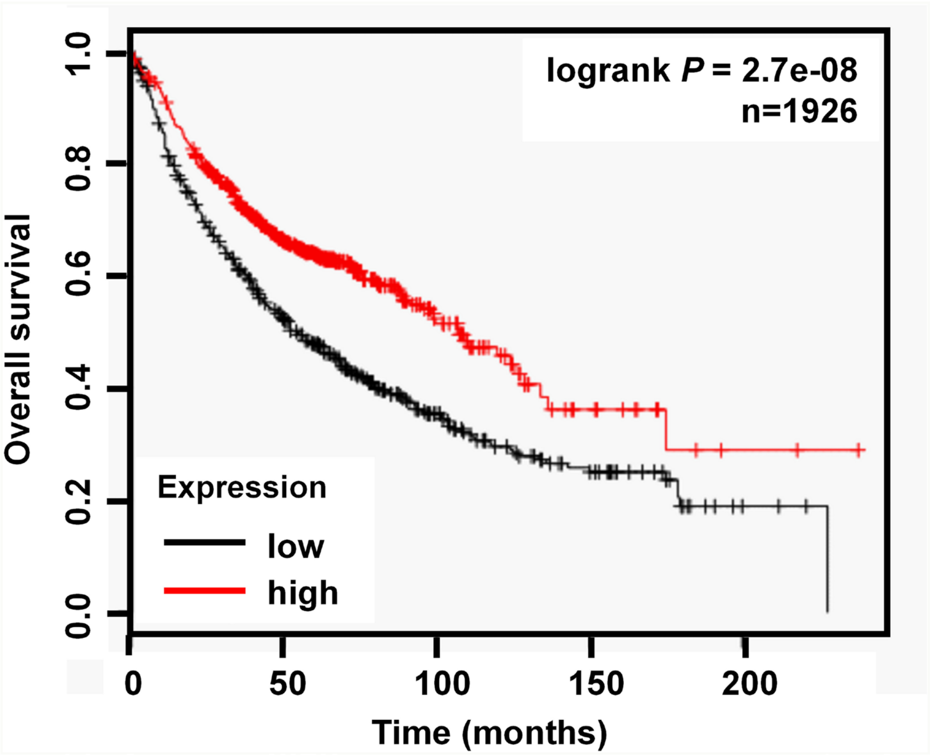

Supplementary Figure 2: QKI mRNA expression correlates with the prognosis of LC patients. Kaplan-Meier analysis of the correlation between the level of *QKI* mRNA and overall survival of 1926 LC patients from the Kaplan Meier plotter (http://www.kmplot.com).

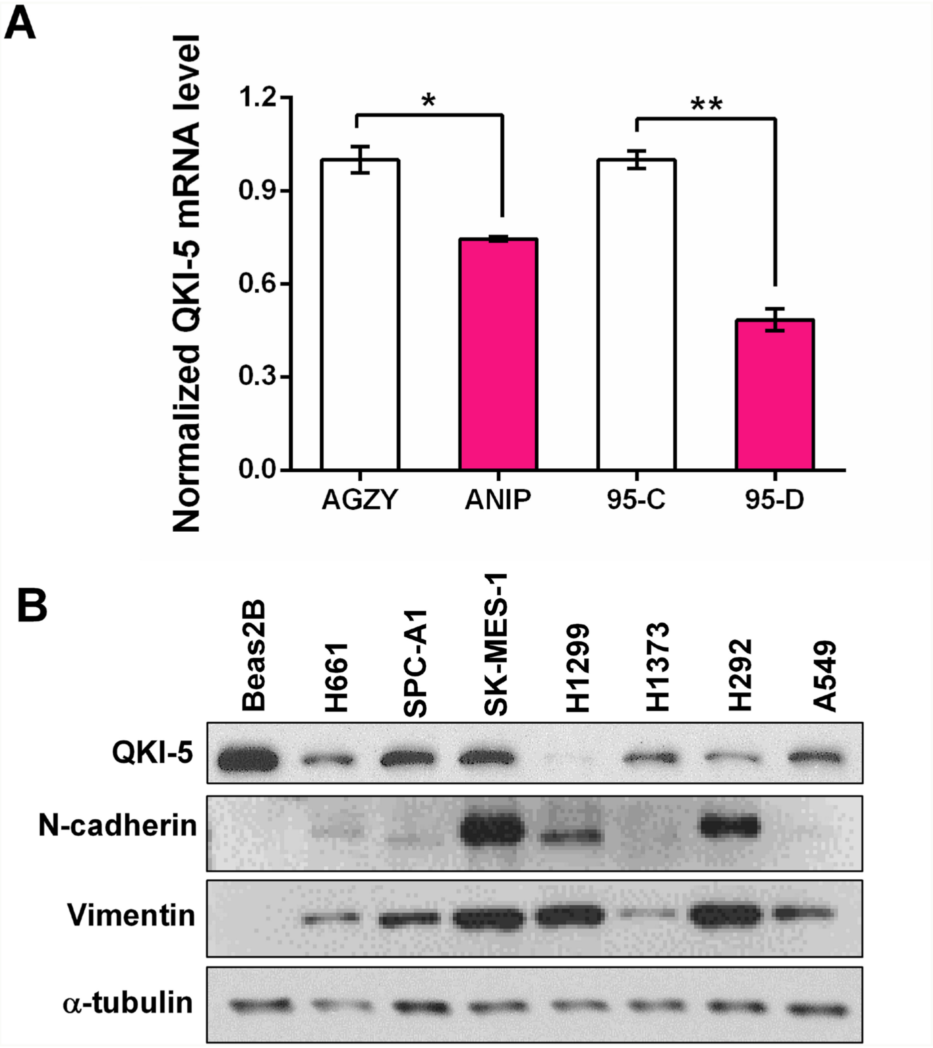

Supplementary Figure 3: Expression of QKI-5 is decreased in LC cell lines. (A) *QKI-5* mRNA expression in two pairs of LC cell lines with different metastatic potentials (AGZY vs ANIP, 95C vs 95D). The data are presented as the mean ± SD. *P<0.05, **P<0.01. **(B)** Western blotting analyses of QKI-5 and mesenchymal markers (N-cadherin, Vimentin) expressions in a panel of LC cell lines and the bronchial epithelial cell (Beas2B). α-tubulin was used as the loading control.

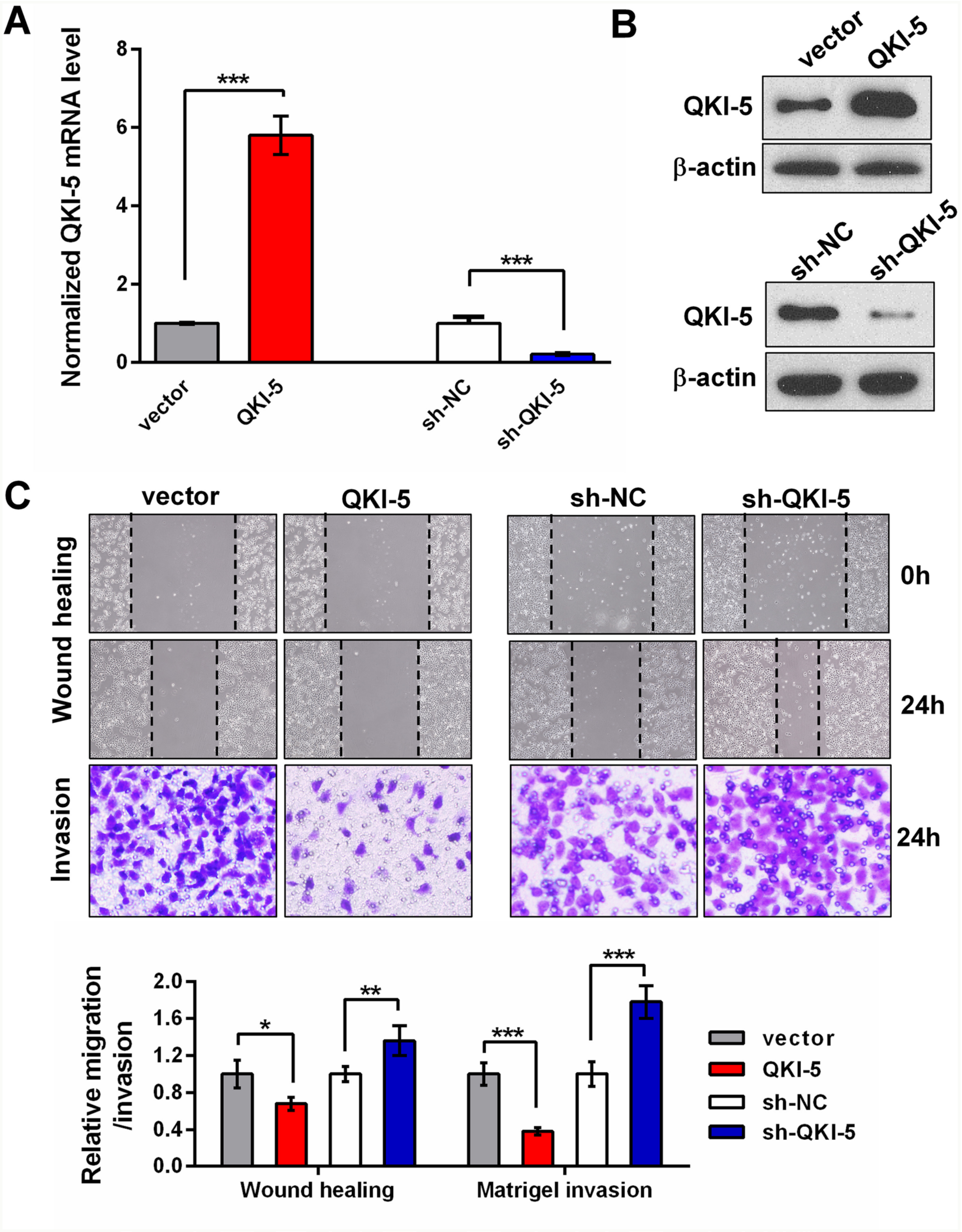

Supplementary Figure 4: The inhibitory effects of QKI-5 on the migration and invasion of SPC-A1 cells. (**A** and **B**) Detections of qRT-PCR **(A)** and Western blotting **(B)** of QKI-5 overexpression and knockdown efficiencies in stable SPC-A1 subcell lines constructed with the indicated viruses or plasmids. The *QKI-5* mRNA level was normalized against *GAPDH*. The ratios of *QKI-5*/*GAPDH* in the vector and sh-NC groups were arbitrarily set to 1.0. **(C)** Migration and invasion abilities of the indicated SPC-A1 subcell lines assessed by wound-healing and transwell assays. All the experiments were performed at least in triplicate and the data in A and C are presented as the mean ± SD. **P*<0.05, ***P*<0.01, ****P*<0.001.

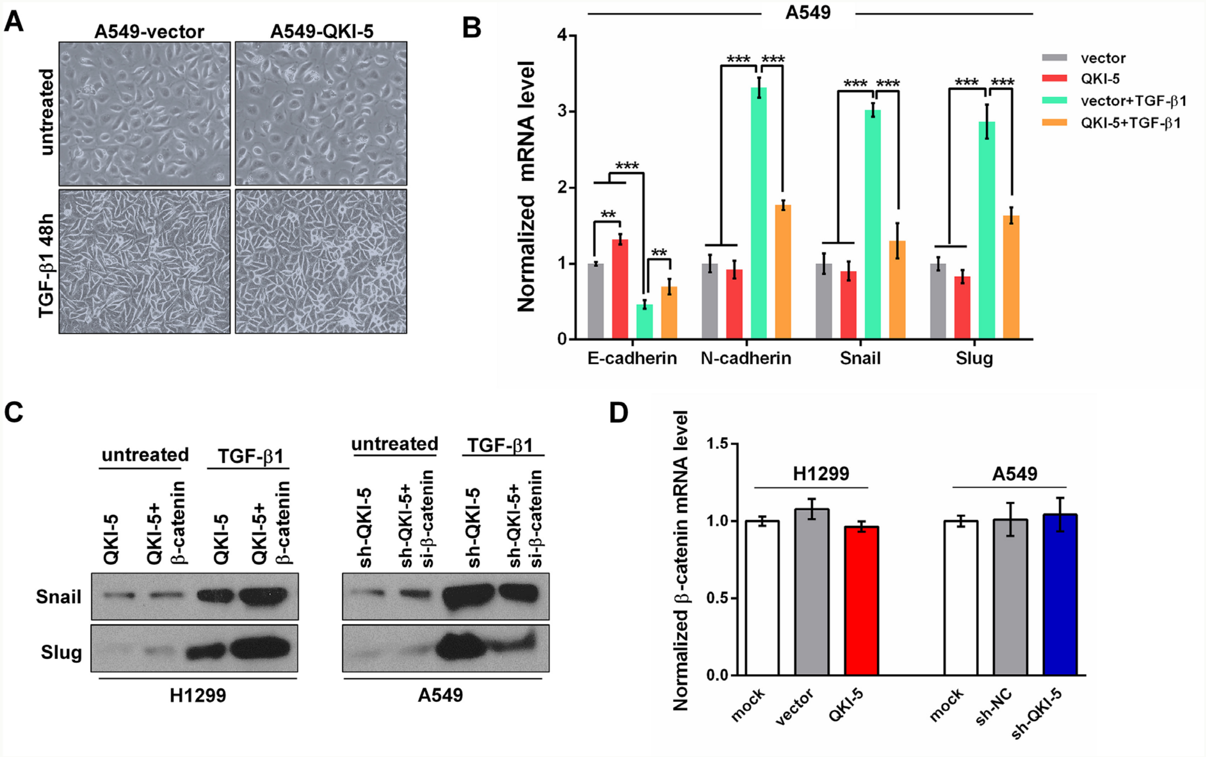

Supplementary Figure 5: QKI-5 overexpression inhibits the TGF-β1-induced EMT of LC cells via interdicting β-catenin signaling pathway. (A) Representative images of A549-vector (control) and A549-QKI-5 (QKI-5-overexpressing) cells untreated or incubated with TGF-β1 (5 ng/mL) for 48 h (×200). **(B)** mRNA levels of *E-cadherin*, *N-cadherin*, *Snail* and *Slug* in the indicated cells incubated with TGF-β1 or untreated in DMEM for 72 h. **(C)** Protein levels of Snail and Slug determined by Western blotting in the indicated cells. **(D)** *β-catenin* mRNA level in the indicated cells; mock = blank control, sh-NC = Scr control, sh-QKI-5 = QKI-5 silence with shRNA. The mRNAs were detected by qRT-PCR and normalized against *GAPDH*. The ratios of the above mRNAs to *GAPDH* mRNA in mock cells were arbitrarily set to 1.0 and the data in B and D are presented as the mean ± SD. $^{**}P<0.01$, $^{***}P<0.001$.

Supplementary Table 1: Clinical characteristics of all tested tissue samples

See Supplementary File 1

Supplementary Table 2: Sequences of RT-PCR and MSP primers

Name	Sequences
**RT-PCR primers**	
**QKI exon5-Forward-1**	5'-GGAGAAGACAGCCTGAAGAAGA-3'
**QKI exon7-Reverse-1**	5'-GTAAGGATGGACACGCATATCG-3'
**QKI exon5-Forward-2**	5'-AAGGAGAAGACAGCCTGAAGAA-3'
**QKI exon7-Reverse-2**	5'- TGGTAAGGATGGACACGCATAT-3'
**β-catenin RIP-Forward**	5'-CAGCTGTATTGTCTGAACTTGC-3'
**β-catenin RIP-Reverse**	5'-CACTTCTTGAGTCACTCCCAAA-3'
**MSP primers**	
**QKI5 MSP-M-Forward**	5'-TAATAAGATCGTTTTTTTAGAGGCG-3'
**QKI5 MSP-M-Reverse**	5'- CCGTAACTTCTTTCGATATTCGTA-3'
**QKI5 MSP-U-Forward**	5'- GGTGTAATAAGATTGTTTTTTTAGAGGT-3'
**QKI5 MSP-U-Reverse**	5'- CCATAACTTCTTTCAATATTCATA-3'

Supplementary Table 3: Sequences of qRT-PCR primers

Name	Sequences
**QKI-5-Forward**	5’-AGCTGCCCTGCGTACTCCTA-3’
**QKI-5-Reverse**	5’-TGGGTGAGGAGTTCCGTTTG-3’
**β-actin-Forward**	5’-GATCATTGCTCCTCCTGAGC-3’
**β-actin-Reverse**	5’-ACTCCTGCTTGCTGATCCAC-3’
**E-cadherin-Forward**	5’-TTCCCTCGACACCCGATTC-3’
**E-cadherin-Reverse**	5’-GTCCCAGGCGTAGACCAAGA-3’
**N-cadherin-Forward**	5’-CCCCTTCACCCAACATGTTT-3’
**N-cadherin-Reverse**	5’-GTGGGATTGCCTTCCATGTC-3’
**Vimentin-Forward**	5’-GAACGCCAGATGCGTGAAAT-3’
**Vimentin-Reverse**	5’-CAGGCGGCCAATAGTGTCTT-3’
**Snail-Forward**	5’-ACCCCAATCGGAAGCCTAAC-3’
**Snail-Reverse**	5’-CGTAGGGCTGCTGGAAGGTA-3’
**GAPDH-Forward**	5’-CCACCCATGGCAAATTCCATGGCA-3’
**GAPDH-Reverse**	5’-TCTAGACGGCAGGTCAGGTCCACC-3’
**β-catenin-Forward**	5’-GGCTGGTGACAGGGAAGACAT-3’
**β-catenin-Reverse**	5’-TTCTGGGCCATCTCTGCTTCT-3’

Supplementary Table 4: Sequences of QKI-5 cloning primers

Name	Sequences
**pcDNA3.1-HindIII-Forward**	5'-CCCAAGCTTATGGTCGGGGAAATGGAAACG-3'
**pcDNA3.1-BamHI-Reverse**	5'-CGCGGATCCTTAGTTGCCGGTGGCGGCT-3'
**pCMV-tag2b-BamHI-Forward**	5'-CGCGGATCCATGGTCGGGGAAATGGAAAC-3'
**pCMV-tag2b-HindIII-Reverse**	5'-CCCAAGCTTTTAGTTGCCGGTGGCGGCT-3'
**pMSCV-puro-BglII-Forward**	5'-GGAAGATCTATGGTCGGGGAAATGGAAACG-3'
**pMSCV-puro-EcoRI-Reverse**	5'-CCGGAATTCTTAGTTGCCGGTGGCGGCT-3'

Supplementary Table 5: Sequences of shRNAs

Name	Sequences
**sh-NC-Sense**	5'-ccggTTCCTGGAACAATTGCTTTTACTCGAGTAAAAG CAATTGTTCCAGGAATTTTTg-3'
**sh-NC-Antisense**	5'-aattcAAAAATTCCTGGAACAATTGCTTTTACTCGAGT AAAAGCAATTGTTCCAGGAA-3'
**sh-QKI-5-Sense**	5'-ccggAAGCACCTACAGAGATGCCAACTCGAGTTGGCA TCTCTGTAGGTGCTTTTTTTg-3'
**sh-QKI-5-Antisense**	5'-aattcAAAAAAAGCACCTACAGAGATGCCAACTCGAGT TGGCATCTCTGTAGGTGCTT-3'

Supplementary Table 6: Sequences of siRNAs

Name	Sequences
**si-β-catenin-Sense**	5'-CCCACUAAUGUCCAGCGUUdTdT-3'
**si-β-catenin-Antisense**	5'-AACGCUGGACAUUGUGGGAdTdT-3'
**si-NC-Sense**	5'-GGUGGAACAAUUGCUUUUAdTdT-3'
**si-NC-Antisense**	5'-UAAAAGCAAUUGUUCCACCdTdT-3'
