# Supplementary material for: Quaking-5 suppresses aggressiveness of lung cancer cells through inhibiting β-catenin signaling pathway
Source: Oncotarget. 2017 Jul 7;8(47):82174–84. doi: 10.18632/oncotarget.19066 (PMC5669880; doi:10.18632/oncotarget.19066)
Supplement: Supplementary file 2 [file oncotarget-08-82174-s002.docx]

**Supplementary Table 1. Clinical characteristics of all tested tissue samples**

| **No.** | | **Gender** | | **Age** | | **Histology** | | **Smoking history** | | **LNM status** | | **Stage** | |
| --- | --- | --- | --- | --- | --- | --- | --- | --- | --- | --- | --- | --- | --- |
| 1 | | M | | 48 | | IP | | S | | - | | - | |
| 2 | | M | | 55 | | IP | | S | | - | | - | |
| 3 | | F | | 51 | | IP | | N | | - | | - | |
| 4 | | M | | 62 | | IP | | S | | - | | - | |
| 5 | | M | | 50 | | LB | | S | | - | | - | |
| 6 | | F | | 48 | | LB | | N | | - | | - | |
| 7 | | F | | 51 | | LB | | S | | - | | - | |
| 8 | | M | | 60 | | LB | | S | | - | | - | |
| 9 | | F | | 52 | | IP | | S | | - | | - | |
| 10 | | M | | 45 | | IP | | S | | - | | - | |
| 11 | | M | | 60 | | IP | | N | | - | | - | |
| 12 | | F | | 44 | | IP | | S | | - | | - | |
| 13 | | M | | 53 | | IP | | N | | - | | - | |
| 14 | | M | | 65 | | IP | | S | | - | | - | |
| 15 | | M | | 52 | | IP | | N | | - | | - | |
| 16 | | F | | 51 | | IP | | N | | - | | - | |
| 17 | | M | | 49 | | LB | | S | | - | | - | |
| 18 | | F | | 52 | | LB | | S | | - | | - | |
| 19 | | M | | 59 | | LB | | S | | - | | - | |
| 20 | | F | | 47 | | LB | | N | | - | | - | |
| 21 | | M | | 65 | | SCC | | Sr | | - | | II | |
| 22 | | M | | 75 | | ADC | | S | | - | | I | |
| 23 | | F | | 59 | | ADC | | N | | - | | II | |
| 24 | | F | | 65 | | ADC | | S | | - | | II | |
| 25 | | M | | 60 | | SCC | | N | | - | | I | |
| 26 | | F | | 59 | | ADC | | N | | - | | III | |
| 27 | | M | | 49 | | SCC | | S | | - | | I | |
| 28 | | M | | 54 | | SCC | | S | | - | | I | |
| 29 | | F | | 62 | | ADC | | N | | - | | II | |
| 30 | | F | | 58 | | ADC | | S | | - | | II | |
| **No.** | | **Gender** | | **Age** | | **Histology** | | **Smoking history** | | **LNM status** | | **Stage** | |
| 31 | | M | | 64 | | ADC | | S | | - | | III | |
| 32 | | F | | 53 | | SCC | | S | | - | | II | |
| 33 | | M | | 59 | | ADC | | S | | - | | II | |
| 34 | | M | | 52 | | ADC | | S | | - | | I | |
| 35 | | F | | 60 | | ADC | | N | | - | | II | |
| 36 | | M | | 64 | | SCC | | N | | - | | I | |
| 37 | | F | | 59 | | ADC | | S | | - | | II | |
| 38 | | M | | 53 | | SCC | | N | | - | | II | |
| 39 | | M | | 66 | | ADC | | S | | - | | I | |
| 40 | | M | | 68 | | SCC | | S | | - | | III | |
| 41 | | M | | 74 | | SCC | | S | | + | | III | |
| 42 | | F | | 55 | | ADC | | S | | + | | IV | |
| 43 | | M | | 79 | | ADC | | N | | + | | II | |
| 44 | | M | | 65 | | SCC | | S | | + | | IV | |
| 45 | | M | | 57 | | SCC | | N | | + | | III | |
| 46 | | M | | 43 | | ADC | | S | | + | | III | |
| 47 | | F | | 61 | | ADC | | S | | + | | II | |
| 48 | | M | | 64 | | SCC | | S | | + | | III | |
| 49 | | M | | 66 | | SCC | | N | | + | | IV | |
| 50 | | F | | 58 | | ADC | | S | | + | | III | |
| 51 | | M | | 71 | | ADC | | N | | + | | III | |
| 52 | | M | | 63 | | ADC | | S | | + | | II | |
| 53 | | M | | 60 | | SCC | | S | | + | | III | |
| 54 | | M | | 64 | | ADC | | S | | + | | IV | |
| 55 | | F | | 56 | | ADC | | S | | + | | III | |
| 56 | | M | | 59 | | ADC | | N | | + | | III | |
| 57 | | F | | 72 | | ADC | | S | | + | | II | |
| 58 | | M | | 62 | | SCC | | S | | + | | III | |
| 59 | | M | | 63 | | ADC | | S | | + | | IV | |
| 60 | | M | | 57 | | ADC | | S | | + | | III | |

**Notes:** Expansions for F: Female, M: Male, IP: inflammatory pseudotumor, LB: lung bullous, SCC: Squamous cell carcinoma, ADC: Adenocarcinoma, S: Smoker, N: Nonsmoker.
